# How AI should be used in radiology: assessing ambiguity and completeness of intended use statements of commercial AI products

**DOI:** 10.1186/s13244-024-01616-9

**Published:** 2024-02-16

**Authors:** Kicky G. van Leeuwen, Dennis M. Hedderich, Hugh Harvey, Steven Schalekamp

**Affiliations:** 1 Department of Medical Imaging, Radboudumc, P.O. Box 9101, Nijmegen, 6500 HB The Netherlands; 2 AIforRadiology.com, Utrecht, The Netherlands; 3 Scarlet, London, UK; 4 School of Medicine, Department of Diagnostic and Interventional Neuroradiology, Klinikum Rechts Der Isar, Technical University of Munich, Munich, Germany; 5 Hardian Health, Haywards Heath, UK

**Keywords:** Radiology, Artificial intelligence, Medical device legislation, Device approval process, Medical device safety

## Abstract

**Background:**

Intended use statements (IUSs) are mandatory to obtain regulatory clearance for artificial intelligence (AI)-based medical devices in the European Union. In order to guide the safe use of AI-based medical devices, IUSs need to contain comprehensive and understandable information. This study analyzes the IUSs of CE-marked AI products listed on AIforRadiology.com for ambiguity and completeness.

**Methods:**

We retrieved 157 IUSs of CE-marked AI products listed on AIforRadiology.com in September 2022. Duplicate products (*n* = 1), discontinued products (*n* = 3), and duplicate statements (*n* = 14) were excluded. The resulting IUSs were assessed for the presence of 6 items: medical indication, part of the body, patient population, user profile, use environment, and operating principle. Disclaimers, defined as contra-indications or warnings in the IUS, were identified and compared with claims.

**Results:**

Of 139 AI products, the majority (*n* = 78) of IUSs mentioned 3 or less items. IUSs of only 7 products mentioned all 6 items. The intended body part (*n* = 115) and the operating principle (*n* = 116) were the most frequently mentioned components, while the intended use environment (*n* = 24) and intended patient population (*n* = 29) were mentioned less frequently. Fifty-six statements contained disclaimers that conflicted with the claims in 13 cases.

**Conclusion:**

The majority of IUSs of CE-marked AI-based medical devices lack substantial information and, in few cases, contradict the claims of the product.

**Critical relevance statement:**

To ensure correct usage and to avoid off-label use or foreseeable misuse of AI-based medical devices in radiology, manufacturers are encouraged to provide more comprehensive and less ambiguous intended use statements.

**Key points:**

• Radiologists must know AI products’ intended use to avoid off-label use or misuse.

• Ninety-five percent (*n* = 132/139) of the intended use statements analyzed were incomplete.

• Nine percent (*n* = 13) of the intended use statements held disclaimers contradicting the claim of the AI product.

• Manufacturers and regulatory bodies must ensure that intended use statements are comprehensive.

**Graphical Abstract:**

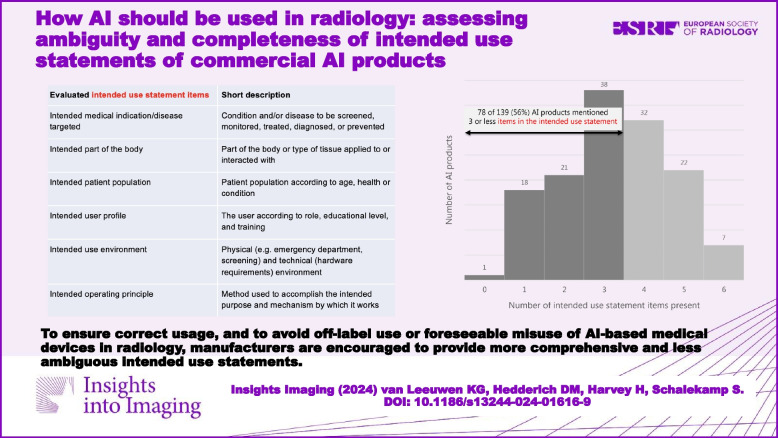

**Supplementary Information:**

The online version contains supplementary material available at 10.1186/s13244-024-01616-9.

## Introduction

Artificial intelligence (AI) products are entering radiology departments with already over 200 CE-marked commercial products available for use in the European Union (EU) [1, 2]. In order to help the radiologist in routine clinical practice, these AI products are designed to help with, e.g., segmentation of healthy tissue and lesions, detection of critical findings and consecutive patient triage, or differential diagnosis of certain disease states [3, 4].

Medical AI applications are considered software as a medical device (SaMD) according to EU legislation and therefore have to comply with the EU Medical Device Regulation (MDR) in order to be marketed and clinically used. The so-called intended use statement (IUS) of SaMD is an important step in the process of compliance and obtaining regulatory clearance, such as a CE mark or FDA clearance [5–8].

In the MDR, the terms intended use and intended purpose are used interchangeably and defined as follows: *‘intended purpose’ means the use for which a device is intended according to the data supplied by the manufacturer on the label, in the instructions for use or in promotional or sales materials or statements and as specified by the manufacturer in the clinical evaluation (Chapter 1, 2.12)*. However, the exact elements that should be part of this intended use statement are not clearly stated in the above-cited MDR definition of the IUS itself but can be found elsewhere in the MDR’s technical documentation (Annex II, 1.1.) [9]. Additional documentation by the International Organization for Standardization (ISO) and the International Electrotechnical Commission (IEC) provides more guidance. For example, ISO 14971:2019 for the application of risk management to medical devices states: “the intended use should take into account information such as the intended medical indication, patient population, part of the body or type of tissue interacted with, user profile, use environment, and operating principle” [10–13].

Unfortunately, as of now, the IUS of CE-marked medical AI products is rarely made publicly available by manufacturers, making it difficult for (potential) users to track down the correct use of their medical device in clinical practice. A critical discussion of intended use statements by radiological societies is hampered by this lack of information. Understanding the intended use of AI products by radiologists is critical to the safe operation of the device, and consequently for patient safety, as underlined by MDR I (43) with the following statement: “Transparency and adequate access to information, appropriately presented for the intended user, are essential in the public interest, to protect public health, to empower patients and healthcare professionals and to enable them to make informed decisions.” Therefore, IUSs of medical AI products need to be comprehensive and clear, without ambiguity. However, this has not been analyzed systematically, yet.

To fill this gap, this study aims to investigate the completeness and potential ambiguity of IUSs for AI products in radiology with respect to the requirements made within the MDR and ISO/IEC standards. Additionally, we make the statements publicly available on www.AIforRadiology.com to increase transparency.

## Methods

### Product inclusion

Inclusion was based on product listings on AIforRadiology.com. This platform maintains a database of CE-marked AI products for radiology. In March 2022, all vendors with listed products (87 vendors, 191 products) were requested to submit or verify an IUS for their product(s). Any new products added to the listing were included until September 26, 2022 (*n* = 12), leading to a total of 203 products. For 46 AI products, the vendor did not respond; therefore, these products were not analyzed. In total, 157 IUSs were collected. Discontinued products (*n* = 3) and duplicate products (*n* = 1) were removed from the analysis. One vendor (Quibim) had 15 biomarker-based products listed with the same intended use. To prevent overrepresentation of this single IUS in the analysis, we included only one product, and the other 14 were excluded. In total, 139 products and their respective IUSs were eligible for analysis. The full list of evaluated AI products is available in the Supplementary materials.

For each product, the CE risk class (I, IIa, IIb, or III) at that time was documented as well as the relevant regulation under which it was certified, either the former EU Medical Device Directive (MDD) (pre-May 2021) or current EU MDR. Products were also classified according to their main function or task (quantification, detection, diagnosis, triage, image enhancement).

### Intended use statement items

Since no clear definition of essential intended use statement items is given in the MDR, we scrutinized the intended use statement for the presence (yes/no) of the following six intended use statement items as described in several relevant ISO/IEC standards [10–13]:

- Intended medical indication
- Intended part of the body or type of tissue applied to or interacted with
- Intended patient population
- Intended user profile
- Intended use environment
- Intended operating principle

Further description of the IUS items is available in Table 1. First, the authors (K.G.v.L., D.M.H., S.S.) scored the same IUS of 30 products, after which a meeting was held to align our definitions of the IUS items and to obtain consensus in case of discrepant scoring. The remaining IUS were scored by one of the authors (K.G.v.L., D.M.H., S.S.), after which a consensus meeting was held to discuss uncertainties in scoring.

**Table 1:** Description of defined item derived from the intended use statements

Item	Short description	Question	Example from intended use statement
Intended medical indication/disease targeted	Condition and/or disease to be screened, monitored, treated, diagnosed, or prevented	What specific medical conditions are you targeting, and what clinical claims are you making?	“The intended use of [PRODUCT] is to perform an automatic determination of bone age and bone health index (BHI).”
Intended part of the body	Part of the body or type of tissue applied to or interacted with	What body part is your product addressing?	“[PRODUCT] indicated for use in the analysis of CT studies of the abdomen.”
Intended patient population	Patient population according to age, health, or condition	What specific patient group is your product aimed at?	“[PRODUCT] uses the screening mammograms of the female population.”
Intended user profile	The user according to the role, educational level, and training	Who are the intended users of your AI solution? What expertise or training is required from these users?	“[PRODUCT] is to be used by qualified medical professionals who are credentialed by their medical institutions to review chest radiographs.”
Intended use environment	Physical (e.g., emergency department, screening) and technical (hardware requirements) environment	Where exactly do you envision your product being used (e.g., on a stand-alone app, integrated)? In what clinical setting (screening, acute care, low-income country, etc.)?	“The device is intended to be used when the patient is not in a life-threatening state of health, time is not critical for medical decisions and no major therapeutic interventions are required.” and/or “[PRODUCT] uses a […] standalone Web application in parallel to the ongoing standard of care image interpretation.”
Intended operating principle	Method used to accomplish the intended purpose and mechanism by which it works	What is the input, computational task, and output of your product? How should the user be using the outcomes (triage, stand-alone, concurrent, etc.)?	“[PRODUCT] is comprised of computer assisted reading tools designed to aid the radiologist in the detection of pulmonary nodules during review of CT examinations […] It is used to aid users in making a final diagnosis and patient management decisions by providing adjunctive information.”
Disclaimers	Warnings and contraindications for use	How should the product be used or not be used? What is foreseeable misuse?	“[PRODUCT] is not an interpretive or diagnostic aid and should be used only as adjunctive information when the final assessment […] is made by an […] interpreting physician.”

Furthermore, it was documented if contra-indications or warnings were made in the IUS. These items were considered disclaimers. Disclaimer statements included wording such as “act per the standard of care” and “not for diagnostic use.” The disclaimers were compared with claims in the IUS, if present. The presence of potential conflicts of claims and disclaimers were categorized as yes/no.

### Analysis

The overall completeness of the IUSs of the AI products was assessed by counting the presence of the IUS items and expressing this as a percentage of the total number of products analyzed. A subanalysis was subsequently performed based on the regulatory risk classification and the main intended task of the product. Similarly, the conflicting claims and disclaimers were counted. A histogram was constructed to show the level of completeness of each product.

IUSs for which each respective vendor agreed to make publicly available can be found on www.AIforRadiology.com.

## Results

### Product inclusion

We received an IUS for 157 out of 203 products, of which 139 were included for analysis.

The included products were certified under the current MDR (class IIa = 23, class IIb = 9) and former MDD (class I = 52, class IIa = 55). Most products were intended for quantification (*n* = 54), detection (*n* = 36), or diagnosis (*n* = 25). A smaller number of products focused on triage (*n* = 18) or image enhancement (*n* = 6).

### Completeness based on intended use statement items

**Fig. 1:**
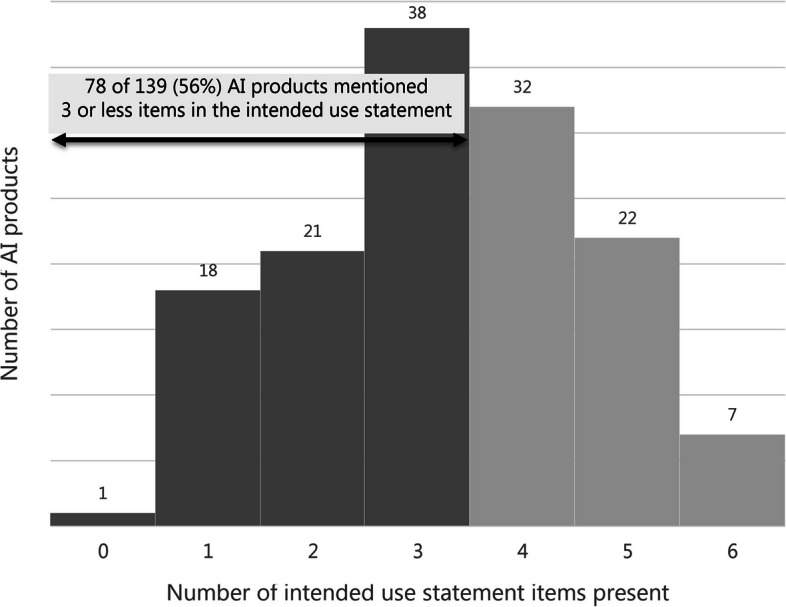
Histogram of the number of items present per intended use statement ranging from 0 to 6 items. Intended use items scored were intended medical indication, intended part of the body or type of tissue applied to or interacted with, intended patient population, intended user profile, intended use environment, and intended operating principle

Only 7 products had an IUS that contained all of the 6 defined items. The majority (*n* = 78) mentioned 3 or less items (0 items, *n* = 1; 1 item, *n* = 18; 2 items, *n* = 21; 3 items, *n* = 38). Four items were mentioned in 32 statements and 5 items in 22 statements (Fig. 1).

**Fig. 2:**
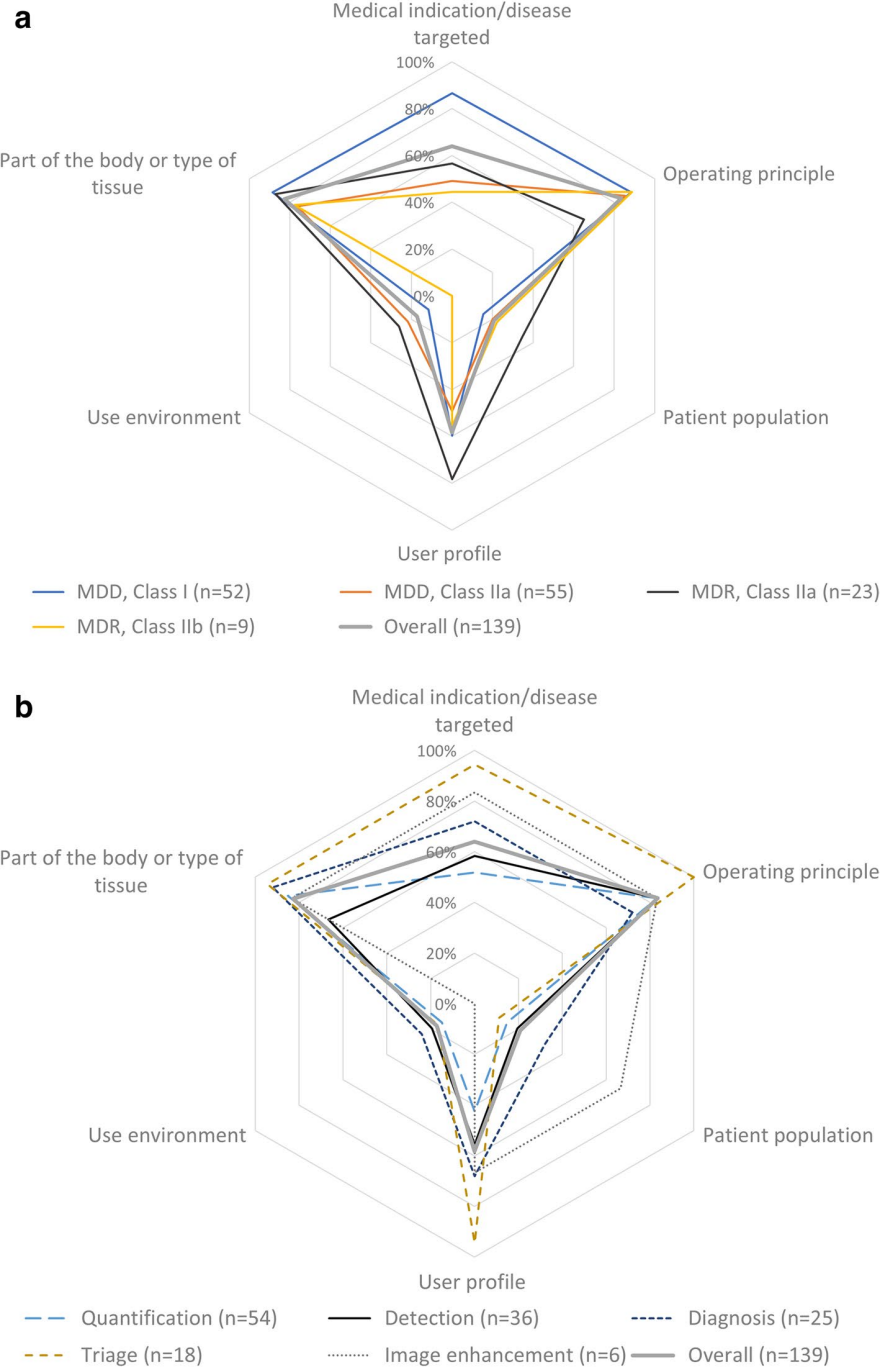
Two spider charts of the percentage of IUS items present per regulatory class of the product (**a**) and product type (**b**). MDD, Medical Devices Directive; MDR, Medical Devices Regulation

Table 2 gives the number of instances each item was present in the IUS. The intended body part (*n* = 115) and the operating principle (*n* = 116) were most often mentioned. The intended use environment (*n* = 24) and the intended population (*n* = 29) were only mentioned scarcely (Fig. 2). Deidentified example statements of good and poor IUSs are given in Table 3.

**Table 2:** Overview of the specific intended use items for all products and per product type and level of certification

	Intended use item						
	Medical indication/disease targeted	Part of the body or type of tissue	Use environment	User profile	Patient population	Operating principle	Total number of products
Total	89 (64%)	115 (83%)	24 (17%)	81 (58%)	29 (21%)	116 (83%)	139
Regulatory class							
MDD, class I	45 (87%)	46 (88%)	6 (12%)	31 (60%)	8 (15%)	46 (88%)	52
MDD, class IIa	27 (49%)	42 (76%)	12 (22%)	27 (49%)	11 (20%)	47 (85%)	55
MDR, class IIa	13 (57%)	20 (87%)	6 (26%)	18 (78%)	8 (35%)	15 (65%)	23
MDR, class IIb	4 (44%)	7 (78%)	0 (0%)	5 (56%)	2 (22%)	8 (89%)	9
Product function							
Quantification	28 (52%)	46 (85%)	8 (15%)	23 (43%)	8 (15%)	45 (83%)	54
Detection	21 (58%)	24 (67%)	7 (19%)	20 (56%)	7 (19%)	30 (83%)	36
Diagnosis	18 (72%)	23 (92%)	6 (24%)	17 (68%)	8 (32%)	18 (72%)	25
Triage	17 (94%)	17 (94%)	3 (17%)	17 (94%)	2 (11%)	18 (100%)	18
Image enhancement	5 (83%)	5 (83%)	0 (0%)	4 (67%)	4 (67%)	5 (83%)	6

*MDD* Medical Devices Directive, *MDR* Medical Devices Regulation

**Table 3:** Examples of two intended use statements which had a score of 6/6 items present (complete) and two examples that had a score of maximal 2/6 items present (incomplete)

Example	Total score	Presence of	Absence of
“[PRODUCT]’s intended use is to assist trained interpreting operators in analysing the breast ultrasound images of patients with soft tissue breast lesions who have been referred for further diagnostic ultrasound examination, either from screening programmes or referred for work up of a suspected breast lesion. It provides a categorical discrete output, which aligns with ACR Breast Imaging-Reporting and Data System (BI-RADS) categories or the U1-U5 Classification System per the European Guidelines for Quality Assurance in Breast Cancer Screening and Diagnosis to help trained interpreting operators improve their overall accuracy as well as reduce inter- and intra-operator variability The software’s PACS function also enables users to review other breast imaging but is not intended to be used to guide diagnostic or management decisions.”	6/6	Indication, body part, environment, user, patient population, and operating principle	/
“[PRODUCT] is indicated for subjects at risk of Alzheimer’s disease dementia on the recommendation of neurology specialists. Radiology specialists with neuroradiological expertise can use [PRODUCT] to support the reporting of brain MRI investigations in these subjects. Neurology specialists can use [PRODUCT] as an aid to diagnosis and prognosis [PRODUCT] software provides the subject level of risk (low risk or high risk) of being affected by or progressing to Alzheimer’s disease dementia within 24 months of the date of the [PRODUCT] processed examination, i.e., the subject’s brain MRI investigation, possibly in combination with a neuropsychological examination performed no earlier than and no later than one month from the brain MRI However, it should be noted that [COMPANY] considers [PRODUCT] as a support to the neurologists in their diagnosis and prognosis, and as a support to the radiology specialists with neuroradiological expertise in their reporting of brain MRI investigations, who have the sole decision making responsibility.”	6/6	Indication, body part, environment, user, patient population, and operating principle	/
“[PRODUCT] provides CAC analysis by segmentation of four main artery (right coronary artery, left main coronary, left anterior descending and left circumflex artery then extracts calcium on coronary artery to provide Agatston score, volume score and mass score by whole and each segmented artery type. Based on the score, provides CAC risk based on age and gender.”	2/6	Body part and operating principle	Indication and environment, operating principle
“[COMPANY] artificial intelligence software for prioritization of radiographs and emphasis of abnormalities.”	1/6	Operating principle	Indication, body part, environment, user, and patient population

### Ambiguity based on conflicting claims and disclaimers

**Fig. 3:**
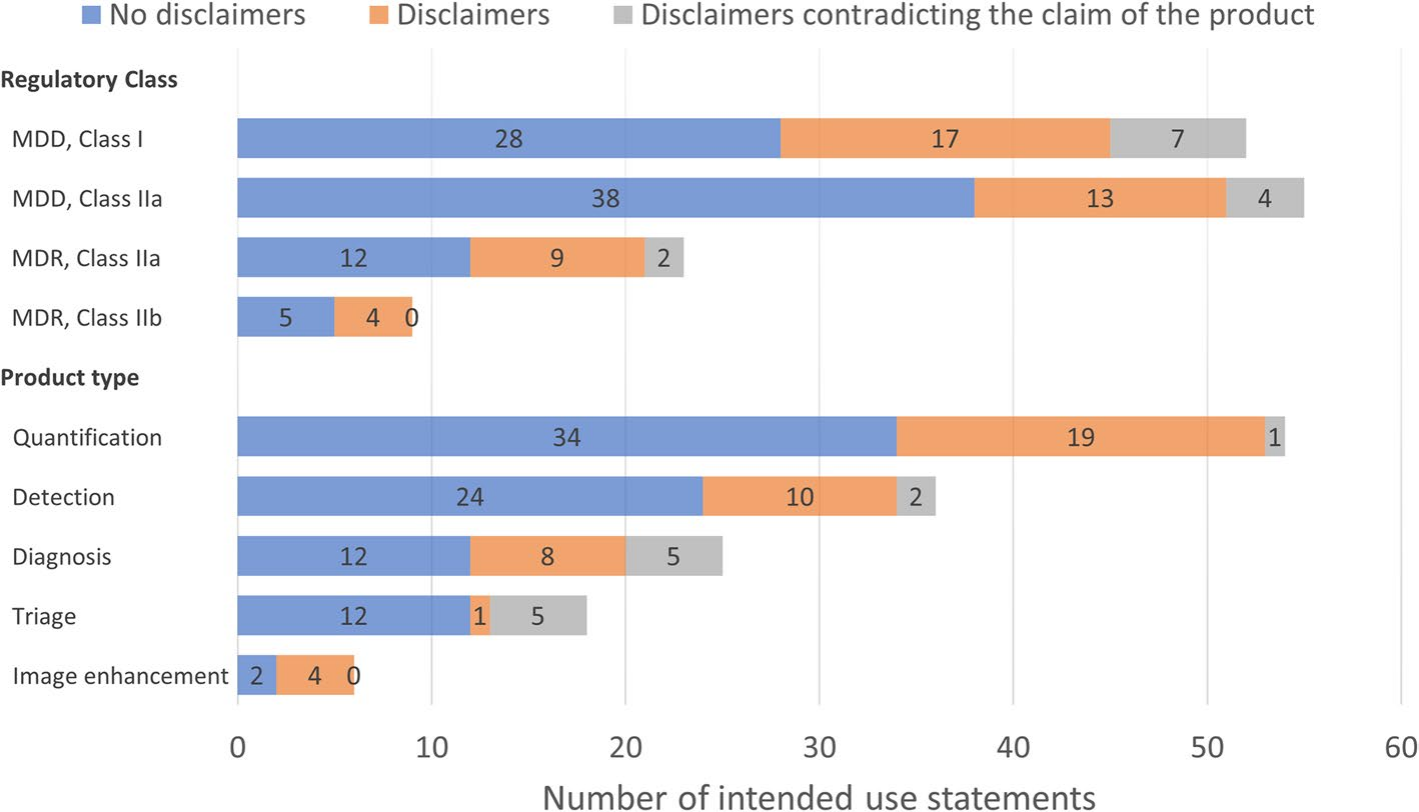
Bar chart of the number of intended use statements without disclaimers (blue), with disclaimers that did not interfere with the claim of the product (orange), and disclaimers that contradict the claim of the product (gray), per regulatory class and product type. MDD, Medical Devices Directive; MDR, Medical Devices Regulation

Of the 139 included statements, 56 held disclaimers. For 13 of these products, the claims and disclaimers were flagged to contradict each other. An overview of the number of disclaimers per product type and regulatory class is shown in Fig. 3. Potential discrepant disclaimer statements included, for example, “act per the standard of care” (*n* = 7) and “not for diagnostic use” (*n* = 6), while claiming to aid in the diagnosis, triaging, or risk scoring of clinical conditions. Example disclaimer statements are available in Table 4.

**Table 4:** Two examples of disclaimers that contradicted the claim in the intended use statement

Examples of disclaimers that contradicted the claim of the AI product
A chest CT AI tool: “It is a tool used to support the oncological workflow by helping the user confirm the absence or presence of lesions, […]. […] it does not directly generate any diagnosis or potential findings.”
A breast density AI tool: “[…]. [PRODUCT] calculates and quantifies a density map and from that determines volumetric breast density […]. [PRODUCT] is not an interpretive or diagnostic aid […]”
A chest X-ray AI tool: “The device […] provides a list of suspected findings on the chest X-ray […] and is able to identify the location of the suspected abnormalities. […] It is not intended to be used as a diagnostic device, a source of medical advice or to aid in determining patient management plan. […] Clinicians are responsible for viewing the original chest X-rays as per the standard of care.”

## Discussion

Many AI products in radiology have an ambiguous or incomplete IUS, as provided to AIforRadiology. Of 139 analyzed products, 78 (56%) were lacking at least 3 essential items in their IUS. Furthermore, 13 (9%) of IUSs held disclaimers that contradicted the claims of the product. The IUS is an important aspect of regulatory documentation as it defines what is considered safe and proper use of the medical device. This is also expressed by the frequent use in the MDR: “Intended purpose” is mentioned a staggering 87 times and “intended use” 31 times. Moreover, the clinical evidence needed to be granted a CE mark is based on the intended use as described in the IUS. Also, it restricts the use of the product by the intended user(s) outside of its intended scope. Therefore, users should be aware that the IUSs of AI products, as provided for this study, were often incomplete and that additional information may be needed to correctly use such AI products in clinical practice.

The intended patient population and use environment were not present in most IUSs. The next most common was a lack of information on the medical indication and the user profile. It is not clear why the intended patient population was only present in 29 of the 139 IUSs. It seems obvious that the intended patient population of an AI product is of utmost importance for safe use in clinical practice, especially since AI systems are prone to bias when trained on a non-representative population. The use environment was only reported in 24 of 139 IUSs and maybe a more difficult item to translate to AI products. However, users should be informed in which (clinical) situation the product is applicable. For instance, an AI product that is designed for screening situations may not be safe to use in a clinical environment.

Our findings are acknowledged by the Medicines & Healthcare products Regulatory Agency (MHRA). This agency mentioned vague intended purposes as a “common issue” and has recently published a guideline to craft IUSs to mitigate this issue for the UK market [14]. These recommendations encourage mentioning explicit items in the IUS that were outlined and used for analysis in this study. We would recommend vendors and regulatory consultants to include all defined IUS items in their public documentation to provide transparent information and to ensure the safe use of AI products in clinical practice, as required by the MDR.

In some of the analyzed statements, we found relevant contra-indications or disclaimers, which are important means to avoid unsafe use, going beyond the actual intended use of the product as a medical device. However, if there is a direct contradiction between the “perceived” intended use of a medical device, e.g., by claims made in the marketing material or suggested in the name of a medical device, disclaimers can add to the confusion about the proper use of a medical device. It is a requirement of all global medical device regulations that marketing material is consistent with regulatory-approved claims and that a device’s labeling covers all externally available marketing material, including manufacturer websites, brochures, and even verbal communications with customers. A recent study already found that 13% of the products were marketed as AI or machine learning, while this was not reflected in the US Food and Drug Administration (FDA) 510 (k) clearance [15]. Further research should focus on how well the intended use in regulatory documents aligns with marketing material and whether disagreement between those two may be a potential source of patient harm.

A clear understanding of the intended use of an AI product is crucial to mitigate the risk of both off-label use and foreseeable misuse. Both vendors and users share the responsibility for the correct and appropriate use of the product. Vendors must take proactive measures to minimize the risk of foreseeable misuse and address potential dangers. On the other hand, users must understand the intended use of the medical device they purchase. Because users, typically radiologists, may be held liable for civil claims and damages in case of any adverse events resulting from the AI product’s misuse [5]. This was made explicit in the example from the USA from April 2022 when the FDA sent a letter to healthcare providers as it became clear that AI products for stroke triage were not used as intended. In a recent statement to healthcare providers, the FDA pointed out that AI-based software tools for the detection of large vessel occlusion in stroke patients need to be used strictly according to their intended use in order to avoid misdiagnosis resulting in patient injury or death [16].

Our study has two main limitations. The first is the subjective nature of analyzing descriptive text. The interpretation of certain IUS elements can be open to debate. Therefore, we tried to be conservative in our analysis. If one of the items was mentioned, but was not entirely clearly stated, we still classified such an item as present. Vague statements were discussed in a panel to overcome inter-reader variability and minimize personal bias. Secondly, the absence of a comprehensive public database containing the IUSs of CE-marked AI products in radiology posed a challenge. Consequently, we had to rely on the information provided by vendors to AIforRadiology. Unfortunately, not all vendors supplied an IUS for their products upon request. Some may have shared a more minimal version of their IUS causing some of the elements to be missing according to our analysis. The European Commission has been developing a publicly accessible database called EUDAMED, in which registering medical devices with complete information will become mandatory by 26 May 2024. However, EUDAMED registration is currently voluntary, and therefore, IUSs for all devices are not yet available.

## Conclusion

In conclusion, our analysis revealed a significant number of AI products listed on AIforRadiology.com with incomplete or ambiguous IUSs. These omissions and/or vague statements, increase the risk of off-label or erroneous application of AI in clinical practice. It is imperative that regulators and central bodies offer comprehensive guidance for the IUS of medical devices including medical AI. Until the enforcement of the EUDAMED database, we encourage manufacturers to be transparent with their regulatory-approved intended use, accurately reflected in all marketing. In addition, we encourage radiologists to get familiar with the intended use, including all its elements, of the AI products they employ, to ensure safe patient care.

### Supplementary Information

**Additional file 1. **List of evaluated AI products.

## Data Availability

The datasets generated during and/or analyzed during the current study are available from the corresponding author upon reasonable request.

## Abbreviations

AI: Artificial intelligence
CE: Conformité Européene (European Union’s mandatory conformity marking)
FDA: US Food and Drug Administration
IEC: International Electrotechnical Commission
ISO: International Organization for Standardization
IUS: Intended use statement
MDD: Medical Devices Directive
MDR: Medical Devices Regulation
